# Adjunctive Aripiprazole Treatment for Risperidone-Induced Hyperprolactinemia: An 8-Week Randomized, Open-Label, Comparative Clinical Trial

**DOI:** 10.1371/journal.pone.0139717

**Published:** 2015-10-08

**Authors:** Jingyuan Zhao, Xueqin Song, Xiaoqing Ai, Xiaojing Gu, Guangbiao Huang, Xue Li, Lijuan Pang, Minli Ding, Shuang Ding, Luxian Lv

**Affiliations:** 1 Department of Psychiatry, Henan Mental Hospital, The Second Affiliated Hospital of Xinxiang Medical University, Xinxiang, China; 2 Henan Key Lab of Biological Psychiatry, Xinxiang Medical University, Xinxiang, China; 3 Department of Psychiatry, The First Affiliated Hospital of Zhengzhou University, Zhengzhou, China; Weill Cornell Medical College Qatar, QATAR

## Abstract

**Objective:**

The present study aimed to evaluate the efficacy and safety of adjunctive aripiprazole treatment in schizophrenia patients with risperidone-induced hyperprolactinemia.

**Methods:**

One hundred and thirteen patients who were receiving a stable dose of risperidone were randomly assigned to either adjunctive aripiprazole treatment (10 mg/day) (aripiprazole group) or no additional treatment (control group) at a 1:1 ratio for 8 weeks. Schizophrenia symptoms were measured using the Positive and Negative Syndrome Scale (PANSS). Rating scales and safety assessments (RSESE, BARS, UKU) were performed at baseline and at weeks 4 and 8. Serum levels of prolactin were determined at baseline and at weeks 2, 4, 6 and 8. Metabolic parameters were determined at baseline and again at weeks 4 and 8.

**Results:**

One hundred and thirteen patients were enrolled in this study, and 107 patients completed the study (54 in the aripiprazole group, and 53 in the control group). PANSS-total scores in the aripiprazole group decreased significantly at week 4 (*P* = 0.003) and week 8 (*P* = 0.007) compared with the control group. PANSS-negative scores in the aripiprazole group also decreased significantly at week 4 (*P* = 0.005) and week 8 (*P*< 0.001) compared with the control group. Serum levels of prolactin in the aripiprazole group decreased significantly at week 2 (*P*< 0.001), week 4 (*P*< 0.001), week 6 (*P*< 0.001) and week 8 (*P*< 0.001) compared with the control group. There were no significant differences in changes of Fasting Plasma Glucose, Total cholesterol, Triglycerides and High Density Lipoprotein within each group at week 4 and 8 execpt low density lipoproteins. There was no significant difference in the incidence of adverse reactions between the two groups.

**Conclusions:**

Adjunctive aripiprazole treatment may be beneficial in reducing serum levels of prolactin and improving negative symptoms in schizophrenia patients with risperidone-induced hyperprolactinemia.

**Trial Registration:**

chictr.org ChiCTR-IOR-15006278

## Introduction

Hyperprolactinemia is a recognized adverse effect of antipsychotic treatment and is associated with acute and chronic clinical consequences in both men and women. The incidence rates of hyperprolactinemia during treatment with antipsychotics are high: 48%–93% in premenopausal women and 42%–47% in men[1]. Women with hyperprolactinemia may display menstrual disturbances, loss of libido, galactorrhea, infertility, hyperestrogenism, and neurological symptoms secondary to macroadenoma. These clinical symptoms are associated with inhibition of the gonadotropic axis[2]. Men may suffer gynecomastia, loss of libido, erectile dysfunction, decrease in ejaculation volume, oligospermia, and infertility[3]. Atypical antipsychotics are widely used as first-line therapy for schizophrenia[4] and risperidone is associated with a greater risk for hyperprolactinemia than other members of the class due to a stronger, more prolonged dopamine D_2_ receptor blockade[5]. Hyperprolactinemia and related adverse reactions are the main reasons for poor treatment compliance in many patients taking risperidone. One clinical strategy to avoid such side effects is to switch from risperidone to another antipsychotic; switching may not, however, be desirable if the patient is responding well to treatment with risperidone[6].

Aripiprazole is another atypical antipsychotic that is widely used for the treatment of schizophrenia. Aripiprazole is a potent partial agonist at dopamine D_2_ receptors, a partial agonist at serotonin 1A (5-HT_1A_) receptors, and an antagonist at serotonin 2A (5-HT_2A_) receptors[7–9]. Given its unique activity profile, aripiprazole could potentially normalize elevated prolactin levels induced by other antipsychotics and some studies have shown that adjunctive aripiprazole treatment can indeed reverse hyperprolactinemia induced by other antipsychotics. In a randomized, double-blind, placebo-controlled study, adjunctive aripiprazole treatment was shown to improve haloperidol-induced hyperprolactinemia in both male and female patients with schizophrenia but to have no significant effects on psychopathology or extrapyramidal symptoms (EPS)[5]. In a separate placebo-controlled study, adjunctive treatment with aripiprazole was shown to reverse hyperprolactinemia induced by risperidone[2]. Another case report suggested, however, that hyperprolactinemia induced by amisulpride is not reversed by adjunctive treatment with aripiprazole[3].

We now report the results of an 8-week randomized, open-label, comparative clinical trial to evaluate the efficacy and safety of adjunctive aripiprazole treatment (10 mg/day) in Chinese schizophrenia patients with risperidone-induced hyperprolactinemia.

## Materials and Methods

### Subjects

All subjects provided written informed consent to participate in the study, which was approved by the Ethics Committee of Henan Mental Hospital, China (Date of the permission by Ethics Committees:August 30, 2012; permission number: 2012LunShen No. 8) and was conducted in accordance with the Declaration of Helsinki[10]. Currently, clinical trial registration is virtually not required for theses randomized, open-label, comparative clinical trials in China, and the trial is allowed to be performed after approval by the institutional ethics review committee. However, we registered the trials in 2015 when we submit the paper (registration number: ChiCTR-IOR–15006278) to meet the international guidelines. The authors confirm that all ongoing and related trials for this drug/intervention are registered. Diagnostic assessments and screening were conducted by a research psychiatrist (J.Z.). The Structured Clinical Interview for Diagnostic and Statistical Manual of Mental Disorders, Fourth Edition (DSM-IV)[11] was used to determine psychiatric diagnoses. Both outpatients and hospitalized patients from Henan Mental Hospital between October 2012 and May 2014 were enrolled in this study. All patients in clinical trials are within the scope of the ethics committee approval time. Inclusion criteria were as follows: i) age 18–45 years; ii) diagnosis of schizophrenia or schizoaffective disorder; iii) stable psychiatric condition; iv) stable on risperidone (4–6 mg/day) for at least 8 weeks; v) elevated serum prolactin level (> 324 mIU/L in males and > 496 mIU/L in females[12]) associated with risperidone treatment; vi) willingness (female subjects of childbearing potential) to practice appropriate birth control methods during the study. Exclusion criteria included: i) inability to provide informed consent; ii) current substance abuse (including alcohol consumption); iii) other significant illnesses including severe cardiovascular, hepatic, or renal disease; iv) history of immunosuppression; v) current or recent radiation or chemotherapy treatment for cancer; vi) pregnancy or breastfeeding; vii) a history of serious adverse reactions to aripiprazole or a history of tardive dyskinesia or neuroleptic malignant syndrome; viii) other conditions (e.g., thyroid or gynecological diseases) that could affect serum prolactin levels. Patients taking medications known to affect glucose tolerance (birth control pills containing norgestrel, steroids, beta-blockers, anti-inflammatory drugs (including aspirin and ibuprofen), thiazide diuretics and valproate sodium) were also excluded from the study; viiii) Patients who were in a special diet or special physical exercise program for weight loss.

### Study design

This was an 8-week randomized, open-label, comparative study designed to evaluate the efficacy and safety of adjunctive aripiprazole treatment in reducing risperidone-induced hyperprolactinemia. After confirmation of eligibility by screening, patients receiving a stable dose of risperidone (4–6 mg/day) were randomly assigned to either adjunctive aripiprazole treatment (10 mg/day) (aripiprazole group) or no additional treatment (control group) at a 1:1 ratio for 8 weeks. Patients were maintained on their usual dose of risperidone and all other medications throughout the study.

### Measures

Schizophrenia symptoms were measured using the Positive and Negative Syndrome Scale (PANSS)[13]. Safety assessments included monitoring adverse events and vital signs as well as physical examination. EPS were assessed using the Rating Scale for Extrapyramidal Side Effects (RSESE) and the Barnes Akathisia Rating Scale (BARS)[14]. General adverse events were evaluated using the Udvalg for Kliniske UndersLgelser (UKU) Side Effect Rating Scale[15]. Safety assessments and ratings were performed at baseline, weeks 4 and 8 by the same investigator (M.D.). Weight (kg) and height (m) were measured, and body mass index (BMI) was calculated for all subjects.

Serum levels of prolactin were determined at baseline and at weeks 2, 4, 6 and 8. Metabolic parameters, including BMI, fasting blood levels of total cholesterol (T-CHO), low density lipoproteins (LDL), high density lipoproteins (HDL), glucose (FPG) and triglycerides (TG), were determined at baseline and at weeks 4 and 8.

Venous blood (10 mL) was collected between 7:00 and 8:00 AM to avoid circadian fluctuations of the analytes. A portion of the blood (5 ml) was allowed to clot at room temperature in a glass tube. Serum was obtained by centrifugation at 3000 rpm for 10 min and stored at − 70°C before use in the prolactin assay. The remaining blood (5 ml) was used to measure blood glucose and lipid levels.

All assays were conducted by analysts blinded to the subjects’ treatment group. Serum levels of prolactin were measured using an electrical chemiluminescence immunoassay (ROCHE e 411, Germany). Glucose oxidase was used to measure plasma glucose levels. Plasma levels of lipids were measured using an enzymatic colorimetric method.

### Statistical analysis

The data were analyzed using SPSS Statistics for Windows version 17 (SPSS Inc., Chicago, IL). Continuous variables were described using means and SD, whereas categorical variables were described using frequencies and percentages. Group comparison was performed using independent t test for continuous variables including Age, duration of illness, Education etc and Chi-square test for categorical variables such as Gender, Smoking, Marriage, Occupation etc. Repeated-measures analyses of covariance (ANCOVA) with treatment regimen (aripiprazole versus control) as a between-group factor, time as a within-group factor and baseline scores as a covariate were used for measures of continuous variables over the 8-week study period. If the overall treatment effect was significant, further between-group contrasts were performed at each time point. Post hoc individual comparisons were made using Least Significant Difference (LSD) test.For all analyses, a *P* < 0.05 (two-tailed) was used to denote statistical significance.

## Results

**Fig 1:**
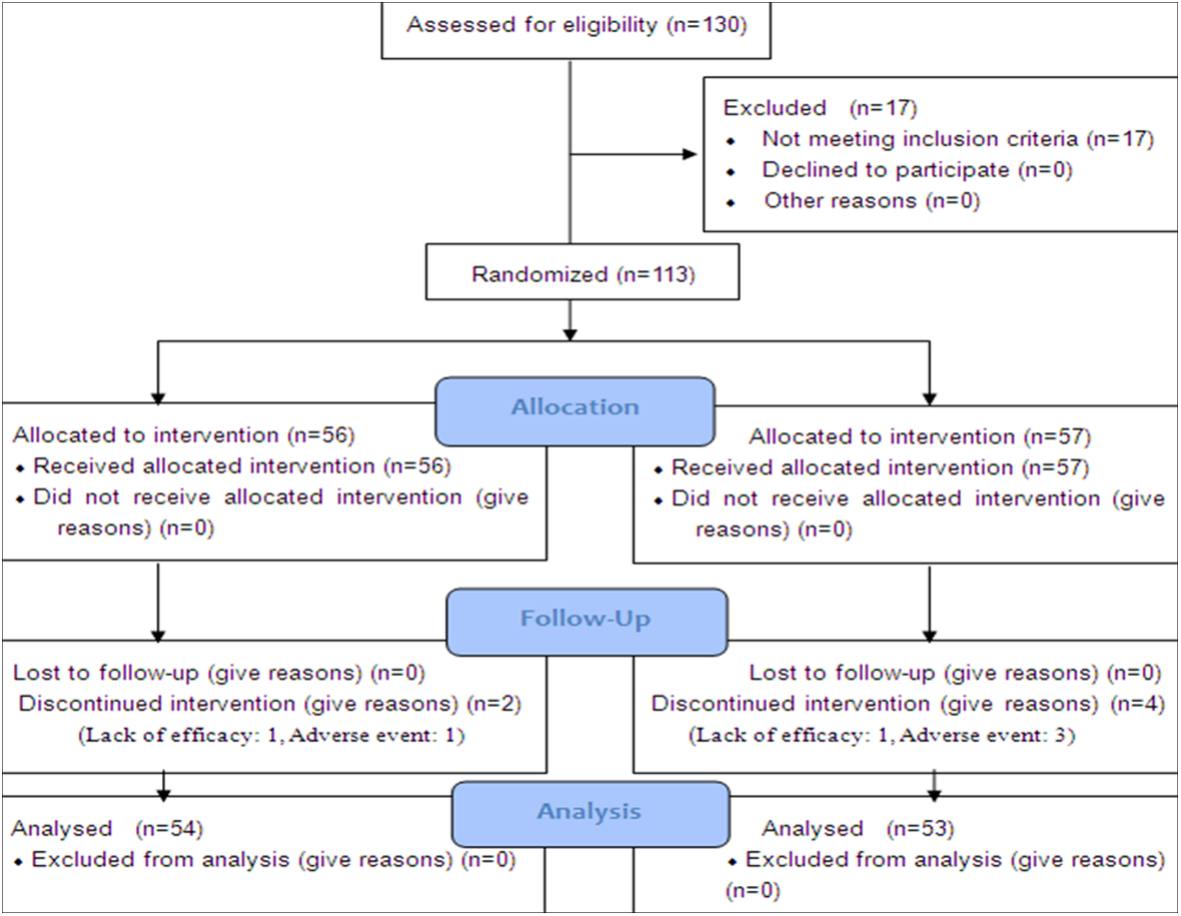
Flow chart of participants through the study.

Out of 130 patients who were screened, 113 patients were enrolled in this study. Individuals were randomly assigned to either the aripiprazole group (N = 56) or the control group (N = 57) in a 1:1 ratio for 8 weeks. There were no significant differences in age, gender, duration of illness, education, smoking status, marital status or occupation between the two groups (*P*> 0.100, Table 1). One hundred and seven patients completed the study, with 54 in the aripiprazole group and 53 in the control group. Six patients withdrew from the study for various reasons before they reached the 8-week time point (Fig 1).

**Table 1:** Demographic and clinical characteristics of study participants. Values are mean ± SD.

Characteristic	Control (N = 57)	Aripiprazole (N = 56)	t or x^2^	*P*
Age (years)	30.41±8.26	28.94±7.84	0.926	0.356
Duration of illness (months)	5.20±5.17	4.18±4.19	1.142	0.257
Education (years)	9.24±3.61	9.96±3.95	-0.941	0.349
*Gender*			0.473	0.491
Male	25(43.86%)	21(37.50%)		
Female	32(56.14%)	35(62.50%)		
*Smoking*			0.832	0.438
Yes	16(28.07%)			
No	41(71.93%)			
*Marriage*			2.934	0.231
Unmarried	25(43.86%)	26(46.43%)		
Married	28(49.12%)	21(37.50%)		
Rests	4(7.02%)	9(16.07%)		
*Occupation*			0.916	0.821
Student	7(12.28%)	6(10.71%)		
Employed	9(15.79%)	11(19.64%)		
Unemployment	16(28.07%)	12(21.43%)		
Peasant	25(43.86%)	27(48.21%)		

### Serum levels of prolactin and metabolic effects

**Fig 2:**
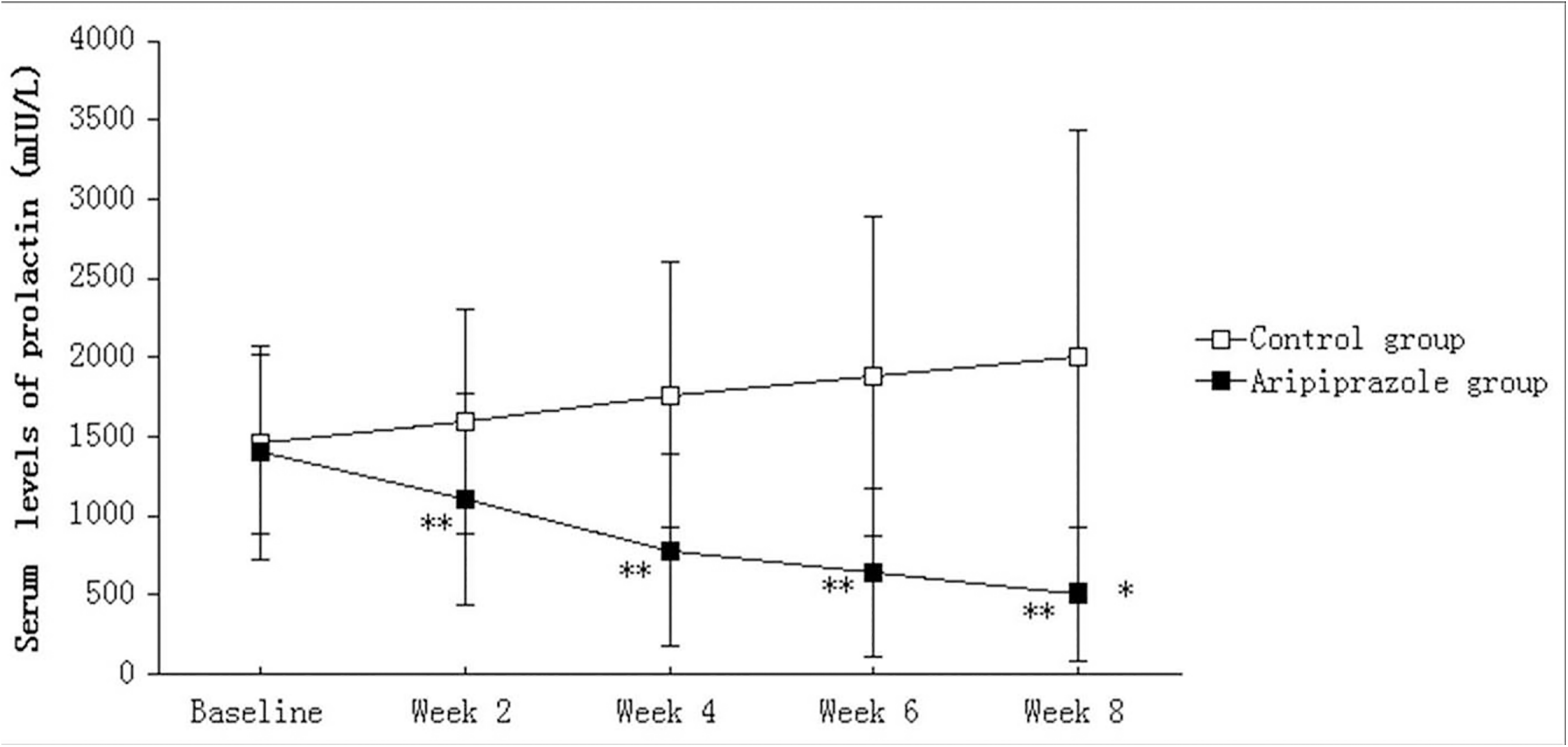
Comparison of serum prolactin levels between groups. Data are expressed as means ± SD and were analyzed by repeated measures analysis of variance or Student's t-test. *P<0.001, Aripiprazole group*vs*. control group for weeks 2–8;** *P*<0.001, *vs*. control.

Repeated-measures ANCOVA on serum levels of prolactin showed a significant treatment-by-time effect [*F*_(1, 105)_ = 35.689, *P*<0.001]. *Post hoc* comparisons of the main effect of treatment revealed aripiprazole group significant differences to control[*F*_(1, 105)_ = 45.183, *P*<0.001]. Further contrast analysis showed that serum levels of prolactin were significantly lower in the aripiprazole group compared with the control group after taking into account baseline values at weeks 2, 4, 6 and 8 (*P*< 0.001) (Fig 2).

**Fig 3:**
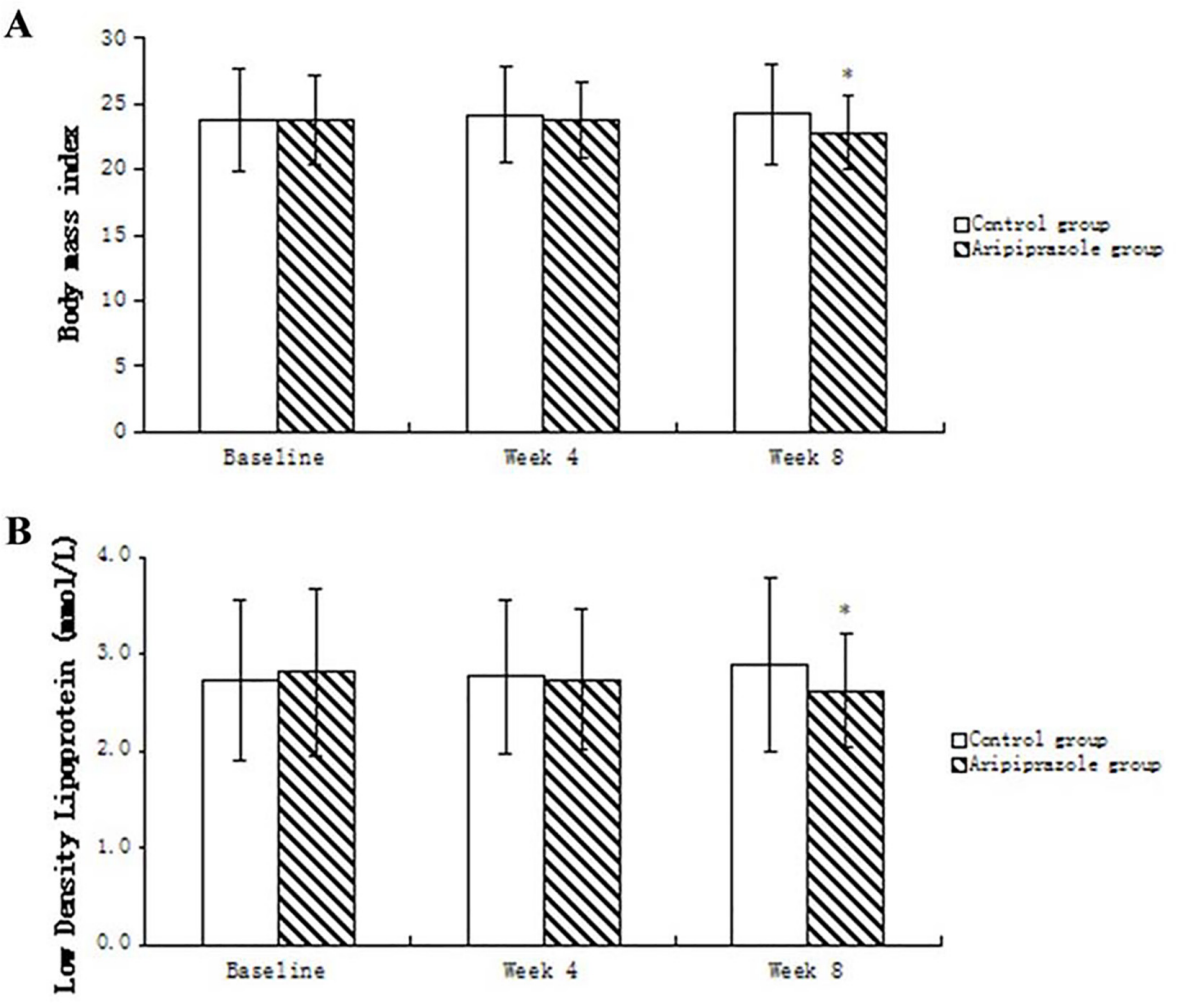
Comparisons of BMIs (A) and plasma LDL levels (B) between groups. Data are expressed as means ± SD and were analyzed byStudent's t-test.*P<0.05, *vs*. control.

Repeated-measures ANCOVA on other metabolic variables showed a significant treatment-by-time effect for BMI [*F*_(1, 105)_ = 13.855, *P*<0.001]or LDL [*F*_(1, 105)_ = 3.143, *P* = 0.045]. Further contrast analysis showed that BMIs or plasma levels of low density lipoproteins were significantly lower in the aripiprazole group compared with the control group after taking into account baseline values at week 8 (*t* = 5.189, *P* = 0.023 or *t* = 4.30, *P* = 0.038, respectively)(Fig 3). There were no significant differences in changes of Fasting Plasma Glucose, Total cholesterol, Triglycerides and High Density Lipoprotein within each group at week 4 and 8(Table 2).

**Table 2:** Changes in plasma glucose, Total cholesterol, Triglycerides,and High Density Lipoprotein after additional treatment aripiprazole. Values are mean ± SD.

	Baseline		Week 4		Week 8	
	Control	Aripiprazole	Control	Aripiprazole	Control	Aripiprazole
Plasma Glucose(mmol/L)	4.87 ±0.61	4.88±0.52	4.90±0.61	4.77±0.65	4.95±0.87	4.75±0.68
Total cholesterol(mmol/L)	4.44 ± 1.08	4.48 ± 1.06	4.50±1.09	4.41±0.90	4.54±1.11	4.32±0.92
Triglycerides(mmol/L)	1.61±0.83	1.69 ±0.87	1.67±0.74	1.59±0.66	1.68±0.70	1.52±0.69
High Density Lipoprotein(mmol/L)	1.32 ±0.43	1.34 ±0.42	1.29±0.43	1.35±0.46	1.28±0.35	1.41±0.43

### Efficacy

**Fig 4:**
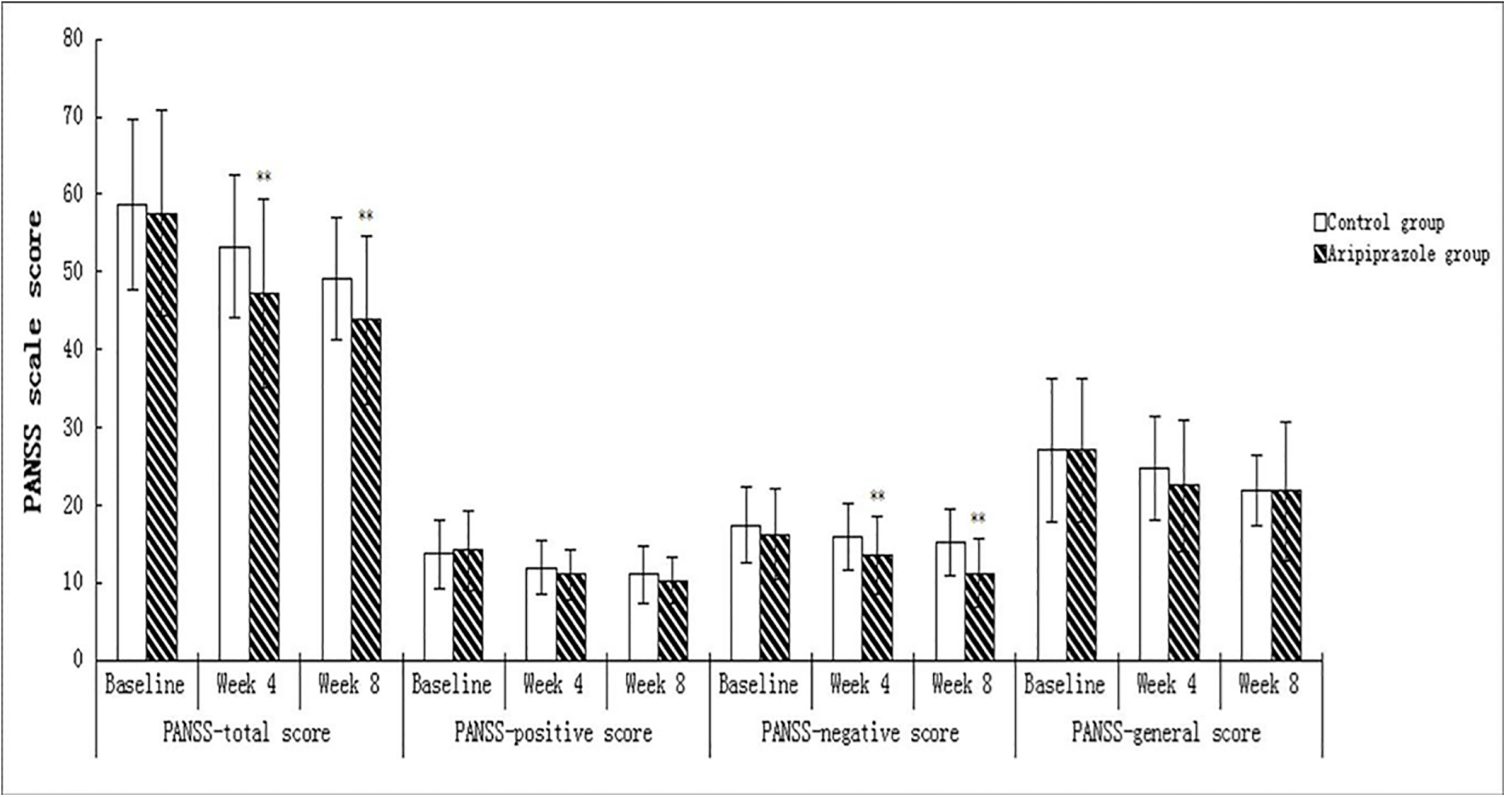
Comparisons of PANSS total and subscale scores between groups. Data are expressed as means ± SD and were analyzed byStudent's t-test. **P <0.01, *vs*. control.

Repeated-measures ANCOVA on the PANSS total and subscale scores showed a significant treatment-by-time effect for the total scores [*F*_(1, 105)_ = 4.123, *P* = 0.045] and the negative subscale scores [*F*_(1, 105)_ = 8.311, *P*<0.005]. Further contrast analysis showed that the total scores and the negative subscale scores were significantly lower in the aripiprazole group compared with the control group after taking into account baseline values at weeks 4 and 8 (*t* = 2.95, *P* < 0.01 and *t* = 4.96, *P* < 0.01, respectively,Fig 4).

### Safety

The incidence of EPS-related adverse events was less than 25% in each group. The incidence of akathisia symptom-related adverse events was less than 10% in each group. There were no significant differences from baseline to endpoint in the RSESE score, the BARS score or the UKU score between the two groups. Ten patients reported nausea in the aripiprazole group but this resolved after about 1 week for most of them; only one patient withdrew from the study (Table 3).

**Table 3:** Adverse events observed in the study (incidence ≥5%).

Event	Control (N = 53) N(%)	Aripiprazole(N = 54) N(%)
At least one event	41(77.4)	45(83.3)
Extrapyramidal events*	19(35.8)	21(38.9)
Akathisia	9(17.0)	9(16.7)
Dry mouth	6(11.3)	8(14.8)
Nausea	3(5.7)	10(18.5)
Somnolence	6(11.3)	5(9.3)
Drooling	7(13.2)	6(11.1)

*Dystonia, muscle rigidity, oromandibular dystonia, parkinsonism, torticollis, and tremor.

## Discussion

The present study shows that adjunctive aripiprazole treatment in schizophrenia patients with risperidone-induced hyperprolactinemia results in a significant reduction in plasma concentrations of prolactin. Our findings are consistent with those of previous reports[6,16,17]. In a placebo-controlled trial carried out in the Republic of Korea, concomitant aripiprazole treatment normalized prolactin levels in 88.5% of patients with haloperidol-induced hyperprolactinemia[5]. Aripiprazole has a higher affinity for dopamine D2 receptors than risperidone. When aripiprazole is administered with risperidone, aripiprazole may thus bind to the dopamine receptor more robustly than risperidone and act as a partial dopamine agonist in the hypodopaminergic state induced by previous risperidone therapy, thereby counteracting hyperprolactinemia.

In our study, adjunctive aripiprazole (10 mg/day) treatment together with a stable dose of risperidone (aripiprazole group), was associated with significant improvements in PANSS total and negative symptom scores compared with the risperidone only group. Previous studies have demonstrated the effectiveness of aripiprazole in treating positive and negative symptoms in patients who started treatment with aripiprazole or switched to aripiprazole from other antipsychotic drugs[18]. The potential benefit of aripiprazole for negative symptoms in patients with schizophrenia is encouraging; further research is needed to explore biomarkers or predictors to identify those who are most likely to respond.

In the present study, adding aripiprazole to a stable dose of risperidone was well tolerated. No significant differences in adverse effects, as measured by the UKU, BARS and RSESE, were seen in the two treatment groups. Our findings were generally consistent with the safety data reported by Kane et al[19].

Some atypical antipsychotics induce metabolic disturbances (e.g. increased body weight, dyslipidaemia and insulin resistance)[20,21] that can lead to increased cardiovascular burden and mortality[22]; weight gain is also a common reason for noncompliance with antipsychotic drugs[23]. Aripiprazole treatment has previously been shown to be associated with a favorable metabolic profile and switching to aripiprazole has led to reductions in body weight[24,25]. The present study showed that plasma levels of LDL were significantly lower in the aripiprazole group compared with the control group after taking into account baseline values at week 8, and no significant differences in other metabolic parameters from baseline to the end of week 8 between the two groups. At the end of week 8, however, the weight of patients in the risperidone only group had increased significantly compared with those receiving adjunctive aripiprazole treatment. This study showed that adjunctive aripiprazole treatment can led to reductions in body weight, but it is likely that our study was not powered to detect the effects of adjunctive aripiprazole treatment on metabolic index changes induced by risperidone.

In conclusion, our study suggests that adjunctive aripiprazole treatment over 8 weeks is safe and well tolerated. It is also effective in reducing prolactin levels elevated by risperidone and may be beneficial in reducing negative symptoms.

There are several limitations to this study. Firstly, the open label design may have introduced unwanted confounding factors into the study. Secondly, the study period of 8 weeks was relatively short to confirm the long-term effect of adjunctive treatment with aripiprazole on hyperprolactinemiaon including menstrual disturbances, galactorrhea, or gynecomastia and metabolic disturbances induced by risperidone. Thirdly, blood levels of risperidone or aripiprazole were not measured. Future studies with larger sample sizes and longer follow up periods are required to confirm our findings.

## Supporting Information

S1 Text CONSORT 2010 Checklist. (DOC)

S2 Text Research Programme (Chinese). (DOC)

S3 Text Research Programme (English). (DOC)
