# Supplementary material for: Adjunctive Aripiprazole Treatment for Risperidone-Induced Hyperprolactinemia: An 8-Week Randomized, Open-Label, Comparative Clinical Trial
Source: PLoS One. 2015 Oct 8;10(10):e0139717. doi: 10.1371/journal.pone.0139717 (PMC4598102; doi:10.1371/journal.pone.0139717)
Supplement: S2 Text — (DOC) [file pone.0139717.s002.doc]

**阿立哌唑口腔崩解片对精神症状及高泌乳**

**素血症影响的研究方案**

**临床调查、观察、检查项目及评估实施时间日程表**

| **观察指标** | **V1**  **第0天** | **2周末（V2）**  **第14天** | **4周末（V3）**  **第28天** | **6周末（V4）**  **第42天** | **8周末（V5）**  **第56天** |
| --- | --- | --- | --- | --- | --- |
| **生命体征** | ★ | ★ | ★ | ★ | ★ |
| **体重、腰围** | ★ | ★ | ★ | ★ | ★ |
| **体重指数** | ★ | ★ | ★ | ★ | ★ |
| **泌乳素** | ★ | ★ | ★ | ★ | ★ |
| **血常规** | ★ |  | ★ |  | ★ |
| **血生化** | ★ |  | ★ |  | ★ |
| **心电图** | ★ |  | ★ |  | ★ |
| **PANSS、CGI评定** | ★ | ★ | ★ | ★ | ★ |
| **RSESE,BARS,UKUAIMS评定** | ★ | ★ | ★ | ★ | ★ |
| **合并用药记录** | ★ | ★ | ★ | ★ | ★ |
| **不良事件记录** | ★ | ★ | ★ | ★ | ★ |
| **月经、泌乳事件记录** | ★ | ★ | ★ | ★ | ★ |
| **性功能异常记录** | ★ | ★ | ★ | ★ | ★ |

附：抽血时间在末次服药后12小时，空腹。泌乳素抽血时间上午10时。

**1.筛选期(第-3～-1天)**

1.1签署知情同意书；

1.2对住院及门诊患者进行筛选，筛选符合入组标准和排除标准的患者。

1.2.1入组标准：①年龄在18~45岁之间；②诊断为精神分裂症或分裂样精神病（DSM-Ⅳ）；③精神症状稳定；④利培酮（4–6 mg/day）已维持8周；⑤利培酮治疗的患者泌乳素高于正常水平(男：> 324 mIU/L ，女：> 496 mIU/L)；⑥由生育能力但无受孕计划的。

1.2.2排除标准：①不能自主签署知情同意的；②有物质或药物滥用的；③有其他严重的躯体疾病的：包括严重心血管、肝脏、肾脏等；④有免疫系统疾病病史的；⑤有癌症病史做过放疗或化疗的；⑥怀孕或哺乳期患者；⑦曾用阿立哌唑有严重不良反应的或不能耐受的；⑧其他可能影响血清泌乳素水平的；病人服用药物已知影响葡萄糖耐量、避孕药含有甲基炔诺酮、类固醇、β受体阻滞剂、抗炎药(包括阿司匹林和布洛芬),噻嗪类利尿剂和丙戊酸钠等也被排除在研究之外。

**2.访视/V1（0天/基线）**

2.1入组评估：符合入组标准和排除标准；

2.2采集人口学信息（性别、年龄、婚姻状况、教育程度、病程、烟酒史等）

2.3采集精神分裂症病史；既往史、个人史、月经生育史、是否存在性功能障碍病史等；

2.4测量生命体征（呼吸、心率、血压、脉搏），体重，腹围，身高等，计算体重指数；进行体格检查及神经系统检查；

2.5实验室检查，包括以下内容：

血常规：红细胞、血红蛋白、白细胞、血小板；

血生化 ：血清泌乳素浓度测定、ALT、AST、尿素氮、肌酐、总胆固醇、甘油三酯、高密度脂蛋白（HDL）、低密度脂蛋白（LDL）、空腹血糖；

心电图；

2.6临床评估，包括以下内容

临床总体印象量表（CGI-I,CGI-S）

阳性与阴性症状量表（PANSS）

锥体外系副反应量表（Rating Scale for Extrapyramidal Side Effects, RSESE）

静坐不能评定量表（BARS）

异常不自主运动量表（AIMS）

不良反应量表（UKU）

2.7治疗情况，包括以下内容：

抗精神病药物治疗情况记录；

伴随用药记录；

不良事件及严重程度记录；

伴随用药记录（详细记录原因及用量）；

月经异常是否存在，性功能障碍是否存在和严重程度评估记录。

2.8利培酮血药浓度监测。

**3.访视/V2（2周末）（第14天）**

3.1测量生命体征（呼吸、心率、血压、脉搏），体重，腹围，身高等，计算体重指数；进行体格检查及神经系统检查；

3.2实验室检查：血清泌乳素浓度测定；

3.3临床评估，包括以下内容

临床总体印象量表（CGI-I,CGI-S）

阳性与阴性症状量表（PANSS）

锥体外系副反应量表（Rating Scale for Extrapyramidal Side Effects, RSESE）

静坐不能评定量表（BARS）

异常不自主运动量表（AIMS）

不良反应量表（UKU）

3.4治疗情况，包括以下内容：

抗精神病药物治疗情况记录；

伴随用药记录；

不良事件及严重程度记录；

伴随用药记录（详细记录原因及用量）；

月经异常是否存在，性功能障碍是否存在和严重程度评估记录。

3.5利培酮血药浓度监测，阿立哌唑血药浓度监测。

**4.访视/V3（4周末）(第28天)**

4.1测量生命体征（呼吸、心率、血压、脉搏），体重，腹围，身高等，计算体重指数；进行体格检查及神经系统检查；

4.2实验室检查，包括以下内容：

血常规：红细胞、血红蛋白、白细胞、血小板；

血生化 ：血清泌乳素浓度测定、ALT、AST、尿素氮、肌酐、总胆固醇、甘油三酯、高密度脂蛋白（HDL）、低密度脂蛋白（LDL）、空腹血糖；

心电图；

4.3临床评估，包括以下内容

临床总体印象量表（CGI-I,CGI-S）

阳性与阴性症状量表（PANSS）

锥体外系副反应量表（Rating Scale for Extrapyramidal Side Effects, RSESE）

静坐不能评定量表（BARS）

异常不自主运动量表（AIMS）

不良反应量表（UKU）

4.4治疗情况，包括以下内容：

抗精神病药物治疗情况记录；

伴随用药记录；

不良事件及严重程度记录；

伴随用药记录（详细记录原因及用量）；

月经异常是否存在，性功能障碍是否存在和严重程度评估记录。

4.5利培酮血药浓度监测，阿立哌唑血药浓度监测。

**5.访视/V4（6周末）(第42天)**

5.1测量生命体征（呼吸、心率、血压、脉搏），体重，腹围，身高等，计算体重指数；进行体格检查及神经系统检查；

5.2实验室检查：血清泌乳素浓度测定；

5.3临床评估，包括以下内容

临床总体印象量表（CGI-I,CGI-S）

阳性与阴性症状量表（PANSS）

锥体外系副反应量表（Rating Scale for Extrapyramidal Side Effects, RSESE）

静坐不能评定量表（BARS）

异常不自主运动量表（AIMS）

不良反应量表（UKU）

5.4治疗情况，包括以下内容：

抗精神病药物治疗情况记录；

伴随用药记录；

不良事件及严重程度记录；

伴随用药记录（详细记录原因及用量）；

月经异常是否存在，性功能障碍是否存在和严重程度评估记录。

5.5利培酮血药浓度监测，阿立哌唑血药浓度监测。

**6.访视/V5（8周末）(第56天)**

6.1测量生命体征（呼吸、心率、血压、脉搏），体重，腹围，身高等，计算体重指数；进行体格检查及神经系统检查；

6.2实验室检查，包括以下内容：

血常规：红细胞、血红蛋白、白细胞、血小板；

血生化 ：血清泌乳素浓度测定、ALT、AST、尿素氮、肌酐、总胆固醇、甘油三酯、高密度脂蛋白（HDL）、低密度脂蛋白（LDL）、空腹血糖；

心电图；

6.3临床评估，包括以下内容

临床总体印象量表（CGI-I,CGI-S）

阳性与阴性症状量表（PANSS）

锥体外系副反应量表（Rating Scale for Extrapyramidal Side Effects, RSESE）

静坐不能评定量表（BARS）

异常不自主运动量表（AIMS）

不良反应量表（UKU）

6.4治疗情况，包括以下内容：

抗精神病药物治疗情况记录；

伴随用药记录；

不良事件及严重程度记录；

伴随用药记录（详细记录原因及用量）；

月经异常是否存在，性功能障碍是否存在和严重程度评估记录。

6.5利培酮血药浓度监测，阿立哌唑血药浓度监测。

**注意事项：**

**观察期禁止合用其他抗精神病药物、禁止合用心境稳定剂、禁止合用抗抑郁剂，若出现则以脱落处理。其他的合并用药应详细记录不良事件、使用原因及剂量，病情转归。**
