# Supplementary material for: Adjunctive Aripiprazole Treatment for Risperidone-Induced Hyperprolactinemia: An 8-Week Randomized, Open-Label, Comparative Clinical Trial
Source: PLoS One. 2015 Oct 8;10(10):e0139717. doi: 10.1371/journal.pone.0139717 (PMC4598102; doi:10.1371/journal.pone.0139717)
Supplement: S3 Text — (DOC) [file pone.0139717.s003.doc]

**Study plan of the effects of aripiprazole orally disintegrating tablets on psychiatric symptoms and hyperprolactinemia**

Implementation schedule of clinical investigation, observation, examination and evaluation

| **Observational parameter** | **V1**  **Day 0** | **2^nd^weekend（V2）**  **Day 14** | **4^th^weekend（V3）**  **Day 28** | **6^th^weekend（V4）**  **Day 42** | **8^th^weekend（V5）**  **Day 56** |
| --- | --- | --- | --- | --- | --- |
| **Vital signs** | ★ | ★ | ★ | ★ | ★ |
| **Weight, waist** | ★ | ★ | ★ | ★ | ★ |
| **BMI** | ★ | ★ | ★ | ★ | ★ |
| **Prolactin** | ★ | ★ | ★ | ★ | ★ |
| **Routine blood test** | ★ |  | ★ |  | ★ |
| **Blood biochemistry** | ★ |  | ★ |  | ★ |
| **ECG** | ★ |  | ★ |  | ★ |
| **PANSS、CGI evaluation** | ★ | ★ | ★ | ★ | ★ |
| **RSESE,BARS,UKUAIMS evaluation** | ★ | ★ | ★ | ★ | ★ |
| **Combinational medication record** | ★ | ★ | ★ | ★ | ★ |
| **Adverse event** | ★ | ★ | ★ | ★ | ★ |
| **Record of abnormal menstrual cycle and lactation events** | ★ | ★ | ★ | ★ | ★ |
| **Record of abnormal sexual function** | ★ | ★ | ★ | ★ | ★ |

Attached: blood sampling was carried out 12 hours after last drug administration, fasting. Blood for prolactin measurement was collected at 10 a.m.

**1. Screening phase (Day -3~Day -1)**

1.1 Sign informed consent;

1.2 Screen inpatient and outpatient, recruiting patients who meet the inclusion and exclusion criteria.

1.2.1 Inclusion criteria were as follows: i) age 18–45 years; ii) diagnosis of schizophrenia or schizoaffective disorder; iii) stable psychiatric condition; iv) stable on risperidone（4–6 mg/day）for at least 8 weeks; v) elevated serum prolactin level (> 324 mIU/L in males and > 496 mIU/L in females) associated with risperidone treatment; vi) willingness (female subjects of childbearing potential) to practice appropriate birth control methods during the study.

1.2.2 Exclusion criteria included: i) inability to provide informed consent; ii) current substance abuse; iii) other signiﬁcant illnesses including severe cardiovascular, hepatic, or renal disease; iv) history of immunosuppression; v) current or recent radiation or chemotherapy treatment for cancer; vi) pregnancy or breastfeeding; vii) a history of serious adverse reactions to aripiprazole or a history of tardive dyskinesia or neuroleptic malignant syndrome; viii) other conditions that could affect serum prolactin levels. Patients taking medications known to affect glucose tolerance (birth control pills containing norgestrel, steroids, beta-blockers, anti-inﬂammatory drugs (including aspirin and ibuprofen), thiazide diuretics and valproate sodium) were also excluded from the study.

**2. Observation/V1 (Day 0/baseline)**

2.1 Recruiting evaluation: meet the inclusion and exclusion criteria.

2.2 Collection of demographic information (gender, age, marital status, education level, course of disease, history of smoking and drinking, etc.)

2.3 Collection of the history of schizophrenia; past history, personal history, menstrual and childbearing history, whether there is a history of sexual dysfunction, etc.;

2.4 measurement of vital signs (breathing, heart rate, blood pressure, pulse), body weight, abdominal circumference, height and others, with body mass index calculated; conducting physical examination and examination of the nervous system;

2.5 Laboratory examinations, including the following:

Routine Blood test: red blood cells, hemoglobin, leukocyte,and platelet;

Blood biochemistry: Serum concentrations of prolactin, ALT, AST, urea nitrogen, creatinine, total cholesterol, triglyceride, high density lipoprotein (HDL), low density lipoprotein (LDL), fasting blood glucose;

ECG

2.6 Clinical evaluation, including:

Clinical general impression scale (CGI-S, CGI-I)

Positive and Negative Syndrome Scale (PANSS)

Rating Scale for Extrapyramidal Side Effects (RSESE)

Barnes Akathisia Rating Scale (BARS)

Udvalg for Kliniske UndersLgelser (UKU) Side Effect Rating Scale

Abnormal involuntary movement scale (AIMS)

2.7 Treatment condition, including:

Record of the efficacy of antipsychotic treatment;

Concomitant medication record;

Record of adverse events and severity;

Concomitant medication records (detailed records of the reasons and dose);

Occurrence of abnormal menstruation, occurrence of sexual dysfunction and the record of the severity assessment.

2.8 monitoring serum concentration of risperidone.

**3. Observation/v2 (2^nd^ weekend) (Day 14)**

3.1 measurement of vital signs (breathing, heart rate, blood pressure, pulse), body weight, abdominal circumference, height and others, with body mass index calculated; conducting physical examination and examination of the nervous system;

3.2 Laboratory examinations: determination of serum prolactin concentration

3.3 Clinical evaluation, including:

Clinical general impression scale (CGI-S, CGI-I)

Positive and Negative Syndrome Scale (PANSS)

Rating Scale for Extrapyramidal Side Effects (RSESE)

Barnes Akathisia Rating Scale (BARS)

Udvalg for Kliniske UndersLgelser (UKU) Side Effect Rating Scale

Abnormal involuntary movement scale (AIMS)

3.4 Treatment condition, including:

Record of the efficacy of antipsychotic treatment;

Concomitant medication record;

Record of adverse events and severity;

Concomitant medication records (detailed records of the reasons and dose);

Occurrence of abnormal menstruation, occurrence of sexual dysfunction and the record of the severity assessment.

3.5 monitoring serum concentrations of risperidone and aripiprazole.

**4. Observation/V3 (4^th^ weekend)(Day 28)**

4.1 measurement of vital signs (breathing, heart rate, blood pressure, pulse), body weight, abdominal circumference, height and others, with body mass index calculated; conducting physical examination and examination of the nervous system;

4.2 Laboratory examinations, including the following:

Routine Blood test: red blood cells, hemoglobin, leukocyte, and platelet;

Blood biochemistry: Serum concentrations of prolactin, ALT, AST, urea nitrogen, creatinine, total cholesterol, triglyceride, high density lipoprotein (HDL), low density lipoprotein (LDL), fasting blood glucose;

ECG；

4.3 Clinical evaluation, including:

Clinical general impression scale (CGI-S, CGI-I)

Positive and Negative Syndrome Scale (PANSS)

Rating Scale for Extrapyramidal Side Effects (RSESE)

Barnes Akathisia Rating Scale (BARS)

Udvalg for Kliniske UndersLgelser (UKU) Side Effect Rating Scale

Abnormal involuntary movement scale (AIMS)

4.4 Treatment condition, including:

Record of the efficacy of antipsychotic treatment;

Concomitant medication record;

Record of adverse events and severity;

Concomitant medication records (detailed records of the reasons and dose);

Occurrence of abnormal menstruation, occurrence of sexual dysfunction and the record of the severity assessment.

4.5 Monitoring serum concentrations of risperidone and aripiprazole.

**5. Observation/V4 (6^th^ weekend)(Day 42)**

5.1 measurement of vital signs (breathing, heart rate, blood pressure, pulse), body weight, abdominal circumference, height and others, with body mass index calculated; conducting physical examination and examination of the nervous system;

5.2 Laboratory examinations: determination of serum prolactin concentration

5.3 Clinical evaluation, including:

Clinical general impression scale (CGI-S, CGI-I)

Positive and Negative Syndrome Scale (PANSS)

Rating Scale for Extrapyramidal Side Effects (RSESE)

Barnes Akathisia Rating Scale (BARS)

Udvalg for Kliniske UndersLgelser (UKU) Side Effect Rating Scale

Abnormal involuntary movement scale (AIMS)

5.4 Treatment condition, including:

Record of the efficacy of antipsychotic treatment;

Concomitant medication record;

Record of adverse events and severity;

Concomitant medication records (detailed records of the reasons and dose);

Occurrence of abnormal menstruation, occurrence of sexual dysfunction and the record of the severity assessment.

5.5 Monitoring serum concentrations of risperidone and aripiprazole.

**6. Observation/V5 (8^th^ weekend)(Day 56)**

6.1 measurement of vital signs (breathing, heart rate, blood pressure, pulse), body weight, abdominal circumference, height and others, with body mass index calculated; conducting physical examination and examination of the nervous system;

6.2 Laboratory examinations, including the following:

Routine Blood test: red blood cells, hemoglobin, leukocyte, and platelet;

Blood biochemistry: Serum concentrations of prolactin, ALT, AST, urea nitrogen, creatinine, total cholesterol, triglyceride, high density lipoprotein (HDL), low density lipoprotein (LDL), fasting blood glucose;

ECG；

6.3 Clinical evaluation, including:

Clinical general impression scale (CGI-S, CGI-I)

Positive and Negative Syndrome Scale (PANSS)

Rating Scale for Extrapyramidal Side Effects (RSESE)

Barnes Akathisia Rating Scale (BARS)

Udvalg for Kliniske UndersLgelser (UKU) Side Effect Rating Scale

Abnormal involuntary movement scale (AIMS)

6.4 Treatment condition, including:

Record of the efficacy of antipsychotic treatment;

Concomitant medication record;

Record of adverse events and severity;

Concomitant medication records (detailed records of the reasons and dose);

Occurrence of abnormal menstruation, occurrence of sexual dysfunction and the record of the severity assessment.

6.5 Monitoring serum concentrations of risperidone and aripiprazole.

**Note:**

**Co-administration of other antipsychotic drugs, mood stabilizer, and antidepressant is prohibited during the observation period. Patients who violate such rule would be excluded from the study. Adverse events, reason of usage, dose and prognosis of the disease related to other co-administrated drugs should be recorded in detail.**
